# The meaning of pharmacological treatment for schizophrenic patients^1^

**DOI:** 10.1590/0104-1169.3427.2466

**Published:** 2014

**Authors:** Kelly Graziani Giacchero Vedana, Adriana Inocenti Miasso

**Affiliations:** 2 PhD, Professor, Escola de Enfermagem de Ribeirão Preto, Universidade de São Paulo, WHO Collaborating Centre for Nursing Research Development, Ribeirão Preto, SP, Brazil

**Keywords:** Schizophrenia, Self Medication, Interpesonal Relations, Patient Satisfaction, Psychotropic Drugs, Medication Adherence

## Abstract

**OBJECTIVE:**

to understand the meaning of medication therapy for schizophrenic patients and formulate a theoretical model about the study phenomenon.

**METHOD:**

a qualitative approach was employed, using Symbolic Interactionism as the theoretical and Grounded Theory as the methodological framework. The research was developed between 2008 and 2010 at three community mental health services in the interior of the State of São Paulo - Brazil. Thirty-six patients and thirty-six family members were selected through theoretical sampling. The data were mainly collected through open interviews and observation and simultaneously analyzed through open, axial and selective coding.

**RESULTS:**

the meaning of the pharmacotherapy is centered on the phenomenon "Living with a help that bothers", which expresses the patients' ambivalence towards the medication and determines their decision making. The insight, access, limitations for self-administration of the drugs and interactions with family members and the health team influenced the patient's medication-related behavior.

**CONCLUSION:**

the theory presented in this study provides a comprehensive, contextualized, motivational and dynamic understanding of the relation the patient experiences and indicates potentials and barriers to follow the medication treatment.

## Introduction

Schizophrenia is a potentially disabling chronic condition that causes a great impact on the patients, families and society. Besides the subjective experience of psychotic symptoms, the disorder affects the individual's quality of life and is associated with significant functional losses^(^ 1 ^)^.

Continuous medication treatment is fundamental to control the symptoms of the disorder^(^ 2 ^)^ when associated with other therapeutic modalities, such as psychotherapy, psychoeducation, sociotherapy, occupational therapy, among others.

The lack of adherence to the pharmacological treatment is associated with the exacerbation of symptoms, worse prognosis, repeated internment, high costs and unnecessary adjustments in the medical prescription^(^ 2 ^)^, justified by a supposed inefficacy of the drug which, in fact, was not used appropriately, which can compromise the patient's safety in the medication treatment.

Patient safety^(^ 3 ^)^ and adherence^(^ 4 ^-^ 6 ^)^ to the pharmacological treatment are important challenges in care practice and require efficient nursing interventions. To plan and implement these actions, the patients' subjectivity, needs, motivations and difficulties need to be considered, more than how precisely they follow the health team's recommendations^(^ 7 ^)^.

In the domestic context, the family serves as a privileged space for care practice and social support, which influence the treatment adherence^(^ 8 ^)^. Patients and family members play a decisive role in the monitoring of the pharmacological treatment.

The construction of a theoretical model about the meaning of the medication treatment for schizophrenia patients permits a comprehensive, contextualized, motivational and empathetic understanding of the reality these individuals experience. It can facilitate the integration between their context, the meaning attributed to the drug therapy, the motivations, decision making and behaviors related to coping with the disorder, besides the identification of potentials and problems to follow the medication therapy.

Thus, this study aimed to understand the meaning of the medication treatment for schizophrenic patients and to build a theoretical model about the study phenomenon.

Symbolic Interactionism was employed as the theoretical framework. This framework presupposes that behavior (observable external act and internal experience) is guided by the individual's definitions of reality. These definitions, in turn, derive from the social interactions in which active individuals exert mutual influence^(^ 9 ^)^.

## Method

A qualitative study was undertaken. Grounded Theory (GT) was used as the methodological framework. The systematic procedures of GT were designated to produce concepts and provide a multivariate and consistent theoretical explanation of the social phenomenon studied^(^ 10 ^)^.

Thirty-six patients and 36 family members were selected to participate in the study, in a theoretical sampling process in which the sample structure is gradually defined during the data collection and simultaneous analysis, as recommended by the GT^(^ 10 ^)^. Three sample groups were constituted, which came from community mental health services that attended to patients with distinct treatment experiences. These services were public and located in the interior of the State of São Paulo - Brazil.

The first sample group consisted of 15 patients and 15 family members from a tertiary psychiatric outpatient clinic that preferably attended to clinically more complex cases. The second sample group included 13 patients and 13 family members followed at a mental health service (secondary level), so as to include people with less problems due to the schizophrenia. In the construction process of the theoretical model, the need emerged for a third sample group, followed at a Psychosocial Care Center, as this service is based on another care model that, besides the medication treatment, includes other therapeutic modalities, psychosocial rehabilitation and users' active participation. The inclusion of participants from distinct services was important to consolidate a more comprehensive theoretical model.

In the construction of the sample, internal variation in the sample groups was sought in terms of personal characteristics and experiences that could influence the construction of the meaning attributed to the medication use, such as: time of diagnosis, gender, age range, education, position in family group, socioeconomic layer, religious belief, medication use, drug administration route, family supervision, among others. This variation in the composition of the groups facilitated the construction of the properties and dimensions in the categories^(^ 10 ^)^.

The criteria to include patients in the study were: being diagnosed with schizophrenia (established by psychiatrist) and taking psychotropic medication(s). The diagnosis was confirmed with the health team and by consulting the patient's history.

The criterion to include the family members in the study was: being mentioned by a schizophrenic patient who participated in the study as the family members most involved in the treatment. Inaptitude to verbally express oneself in Portuguese was used as an exclusion criterion for patients and relatives. The inclusion of family members in this study is justified by their potential to contribute to the understanding of the research phenomenon, as these participants made it possible to confirm and complement information obtained from the patients and the collection of additional information.

Between 2008 and 2010, data collection and analysis were undertaken simultaneously, as recommended by the GT. Recorded open interviews and observation were the main strategies to obtain the data, but were complemented by consultations of patient histories, home visits and case discussion with the health team. The participants could choose to be interviewed at home or in a private environment at the health service.

The first interview held was based on the guiding question: "Tell me what it is like for you to use the medication prescribed by the doctor from the psychiatric service" and, for the relative: "Tell me what it is like for your relative to use the medication prescribed by the doctor from the psychiatric service". The guiding question only directed the aspect that was to be explored. New questions were added to clarify and support the experience.

The research started after receiving approval from the Research Ethics Committee (Process HCRP 10183/2007) and all recommendations for research involving human beings were complied with.

The data analysis process was developed through open, axial and selective coding, in accordance with the premises of the Grounded Theory^(^ 10 ^)^. In the open coding, the data were fragmented into units of meaning, which were mutually compared, considering similarities and differences. This process produced temporary categories and subcategories. In the axial coding, the categories were mutually articulated and each interpretation was taken to the research field for revision or validation. The selective coding resulted in the construction of a theoretical model based on the data.

At the end of the analysis process, all of the study categories were combined around a central category, constituting a paradigm that included: causal conditions (events that influence the occurrence of the phenomenon), phenomenon (central element investigated), context (conditions in which the action/interaction strategies are adopted), intervening conditions (conditions that facilitate or block the strategies adopted), action/interaction strategies (strategies adopted in response to the phenomenon) and consequences (results or expectations related to the action/interaction)^(^ 10 ^)^.

## Results and discussion

The interpretation of the present study data was based on the theoretical framework of Symbolic Interactionism^(^ 9 ^)^. The interpretation of the research phenomenon based on this reference framework departs from the premise that the pharmacological treatment involves individuals in a symbolic interaction. In their interactions, schizophrenia patients attribute meaning to the experience of suffering from the disorder and following the medication treatment. All elements that interfere in the adherence to the drug therapy are defined and redefined in a dynamic and interactional process. The patients' definitions in each situation determine the decision making about the medication treatment.

The analysis of the data collected in this study permitted the construction of a theoretical model centered on the phenomenon "LIVING WITH A HELP THAT BOTHERS", which represents the meaning of the medication treatment for schizophrenia patients.

**Figure 1:**
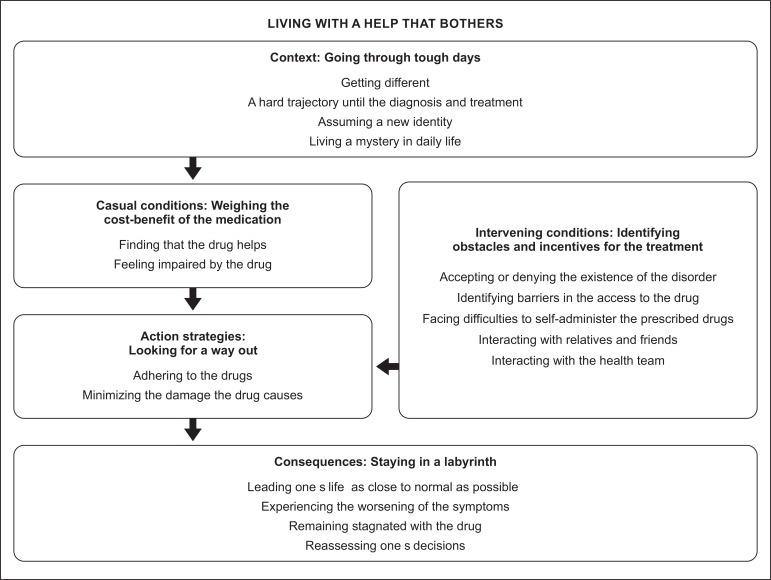
Diagram of the phenomenon: "Living with a help that bothers"

Figure 1 displays a diagram that illustrates the theoretical model about the meaning of the medication treatment for the schizophrenia patients, which will be explained next.

### The Context: Going through tough days

The schizophrenia patients construct the meaning of the medication treatment in a context in which they go through difficult days, marked by symptoms, suffering, limitations, doubts, efforts and concerns. This experience strongly influences their lives.

*I was alone. I don't have a friend, I don't have anything [...] the girls *(who studied at the same school)* all got married, dated and I got like this, no family, nobody [...] I keep watching the others date, sometimes I feel angry. [...] I cannot work for the others anymore, I can't. My head hurts just from watching the others work, drink beer.* (P3)

Schizophrenia negatively influences individuals' quality of life. Besides the subjective experience of the symptoms, it can cause significant functional losses in different spheres of the patients' lives^(^ 11 ^)^.

The symptoms of the disorder drive patients and relatives on a difficult trajectory in the attempt to understand what is happening and seek solutions for the problems they experience.

*It took me five years running after things. And one thinking it was one thing, the other thinking it was something else. And things got uglier and uglier. He used to go to the neurologist, the psychiatrist, each gave one type of medication. But nobody knew what it was. [...] They gave a lot of medication, but it did not work. And that went on until they discovered that he had this. *(F3)

The experiences with the schizophrenia make the patients perceive they are different in the way they are known internally (by themselves) and externally (by other people). Thus, in a painful process, the individual partially takes distance from a previously constructed identity and build a new identity that is depreciated and associated with stigma and limitations.

*The day he had the crisis, it's as if he had died! He was alive in front of me, but it wasn't him. It wasn't him! He was another person. *(F2)

Commonly, the patients experience the schizophrenia more than they understand it. They experience the impact of the schizophrenia concretely, in their daily life, but the disease remains a mystery to some.

*I have a problem in my head. I don't know if it's the neurons that are rotten, or something else. But there's gas coming out of ears on both sides, like a truck with exploding tires, right? The head creates that pressure inside the brain [...] Luckily there's a discharge on both sides of the ears. If it weren't for that discharge I would get barking mad. I already told the doctor, he laughed. [...] Or it's cancer in the head, there's some disgrace. *(P19)

The medication is presented to these people as a resource to eliminate or mitigate the suffering experienced because of the disorder. This context in which the treatment occurs provides relevant elements for the professionals' activities. The literature suggests that peculiarities in the individuals' context and culture can significantly influence the adherence. Interventions to optimize the adherence tend to be more effective when adapted to the individual needs and perceptions about the treatment and articulated with the factors permitting or impeding the adherence^(^ 12 ^-^ 13 ^)^.

### Causal conditions: Weighing the cost-benefit of the medication

The medication provides for the reduction of the symptoms, a better subjective wellbeing, quality of life and relationships, better socialization, activity performance and greater feeling of safety and self-control. Because of these benefits and the symptoms it avoids, the medication gains singular importance in the life of schizophrenic patients.

*Without the medication, perhaps I wouldn't even be alive, you know. I don't know. We don't know our limits. I don't know what I would be capable of doing.* (P10)

The medication is a form of help, however, the patient does not want to need. It represents an imperfect solution, as it does not definitively extinguish the suffering the schizophrenia causes. In addition, the drug entails collateral effects, causes concerns related to the possibility of future damage and represents the obligation to constantly reaffirm the disorder.

The patients aim to definitively get rid of the schizophrenia, without living with the damage the treatment causes. Over time, however, these individuals conclude that they are in a situation in which their desires seem to be incompatible with the reality.

*Ah, we think it's bad, but what can we do? You have to take it.* (P21)

The medication therapy symbolizes the coexistence between the help and the loss. The meaning attributed to the drug reveals the conflict the patients constantly experience throughout the treatment. They simply want to get relief, but are unable to.

*This drug has caused me a lot of evil, but I need it. *(P7)

*It helps in daily life. But there's the side effect too, which bothers in daily life as well. *(P16)

Thus, in following the medication treatment, the schizophrenia patients perceive that they are "LIVING WITH A HELP THAT BOTHERS". In the course of the treatment, their attitude towards the medication use remains biased. The family members underline the bias the patients express towards the medication treatment.

*But there's this: it *(medication)*has the collateral effect that will impair some things, but if you don't take it too, what's it gonna be? If it's good for one thing, I know it will generally cause another damage. But what can you do... *(F5)

*Medication is already called drug, it fixes one thing and ruins another. There's no doubt. *(F6)

Ambivalently, the patient acknowledges that the drug is unpleasant but necessary, and constantly weighs the cost-benefit of the treatment in order to decide on whether to adhere to the medication or not.

Partial adherence and non-adherence are persistent problems among people taking antipsychotic drugs. The lack of adherence is a complex and multifactorial phenomenon. Although non-adherence to the treatment involves factors external to the patient, individual subjectivity is fundamental to maintain treatment in the long term^(^ 13 ^-^ 15 ^)^, in line with the present study.

### Intervening Conditions: Identifying obstacles and incentives for the treatment

When choosing to adhere to or abandon the pharmacological treatment, the patients identify obstacles and incentives for their decision to turn into an effective action.

Among the interviewed patients, some acknowledge suffering from schizophrenia, while others strongly deny this reality. Assimilating the existence of the disorder is important for the drug to be considered a useful and relevant resource for the schizophrenia treatment.

*I do not have that schizophrenia [...] and the medication has not changed anything.* (P4)

*I told the doctor that I don't like to take medication, because, well, I don't like being ill. *(P8)

In the literature, the insight is frequently associated with treatment adherence, although there is no conviction as to whether this association continues in the long term. In addition, the insight is a necessary but insufficient condition for adherence^(^ 16 ^)^.

Another factor that can influence the adherence is medication access. The flaws in the public health system's medication supply, associated with the users' impossibility to purchase the drugs, can compromise the continuity of the drug therapy^(^ 17 ^-^ 18 ^)^.

*I stopped at that time, it was because I really was in no financial conditions. *(P10)

The unintentional behavior of non-adherence to the drug therapy can be favored by the schizophrenia patients' limitations to self-administer the drugs^(^ 17 ^)^, forgetting^(^ 19 ^)^ and complex therapeutic schemes^(^ 18 ^)^.

*That drip remedy is difficult because I can't see. I don't know how many drops fall. *(P19)

*He *(patient)* frequently did not take the medication. He did not pick it up, did not attend the return appointments. *(F9)

Some of these problems are mitigated when the patient gets support from relatives, significant others or health professionals for the sake of medication administration in accordance with their needs^(^ 18 ^)^.

*My father, she and my other brother. These three give the medication, take the medication to him *(patient)* and remind him to take the medication.* (F7)

The family of mental patients need support and preparation to cooperate with the treatment^(^ 20 ^)^, as the family's involvement in the support for the patient is fundamental for a successful treatment^(^ 18 ^)^.

The quality of the bond between the health team and the patient can enhance or impair the maintenance of the drug therapy. A good therapeutic alliance among health professionals, family members and patients provides for better treatment results and reduces the possibility of non-adherence^(^ 6 ^,^ 14 ^,^ 21 ^)^.

*The physician was reliable, then I accepted to take the medication.* (P33)

In the social interaction, the individual shares perspectives, defines the reality, makes decisions and modifies the course of their actions^(^ 9 ^)^. Therefore, interactions can represent opportunities to reconstruct meanings, also with regard to the medication treatment and related behaviors.

Some of the factors that influence the pharmacological adherence can be changed^(^ 2 ^)^. Therefore, they should be considered in the planning of strategic actions to promote the elements that contribute to the medication adherence behavior and to minimize factors that compromise the success of the treatment.

### Action/interaction strategies: Looking for a way out

The schizophrenia patients weight the cost-benefit of the drug in order to select the action strategy they will adopt: adhering to the medication or minimizing the damage the drug treatment causes.

These action strategies represent the search for a way out, for a solution to the conflict of "LIVING WITH A HELP THAT BOTHERS".

*I am going through this, I don't see any way out [...]. I need to try something [...] then I take it* (the drug)*.* (P5)

*If necessary I take it, no problem. What can I do? Give up? If I need it... *(P14)

The assessment about the continuous use of the drug is not static. The patients frequently analyze the suffering the schizophrenia causes, as well as the advantages and disadvantages of the drug, in which sometimes the "help" and sometimes the "loss" prevails. This assessment of the reality and the consequent option to adherence to the medication use or not are part of a dynamic and changeable process. According to Symbolic Interactionism, the meanings are changed in a dynamic process of interpretation the patients accomplish when coping with the situations they experience^(^ 9 ^)^.

*We would like not to want to take it anymore [...] I have taken it *(the medication)*, but I have wanted to quit. *(P6)

In this context, the monitoring and motivation towards adherence should be highlighted^(^ 4 ^,^ 6 ^)^, as the treatment adherence results from a cooperative effort between the health professional and the patient^(^ 7 ^)^.

In some situations of "weighing the cost-benefit of the drug", the patient chooses not to adhere with a view to reducing the damage the pharmacological treatment causes. This is a relevant problem that compromises the treatment success, as the literature appoints that approximately half of the schizophrenia patients do not adhere to the prescribed drugs^(^ 2 ^,^ 17 ^)^.

### Consequences: Staying in a labyrinth

The action strategies the patient adopts can result in the improvement, stabilization or worsening of the clinical conditions of the disorder. The results of these strategies, even when positive, do not fully attend to the patients' expectations. "Staying in a labyrinth" is the way the patients feel when assessing these consequences, as they keep on searching for new exits and solutions. 

*Since I have started the treatment, I should have been discharged by now. If some medicine had worked, I would already have improved, I would already have been cured. It seems as if you're in a labyrinth you're trying to get out of, but then you explode with it. But I'm taking it *(the medication). (P12)

The feeling of being in a labyrinth is experienced in different ways, with varying degrees of satisfaction with the treatment, symptoms, limits and potentials of the schizophrenia patients.

In seeking relief from the adherence to the drug therapy, the patients can reach good levels of functioning and lead a life "as closely to normal as possible". In that context, the individuals do not feel "completely normal" and the drug therapy symbolizes the route to achieve stability and, at the same time, the existence of a limitation.

*I am completely normal, the only problem is that I take medication. *(P22)

*I try to lead my life as closely to normal as possible. So I look for any means I find out there, which can help me. *(P1)

Hence, it is impossible to directly relate the benefits of the medication with satisfaction or adherence. Studies indicate that positive results of drug treatment can favor adherence^(^ 13 ^)^ but, when associated with an expected cure, they can arouse questions about the need to maintain the treatment^(^ 22 ^)^. Therefore, it is not purely the result of the medication treatment that influences the adherence, but the individuals' assessment of this experience.

In some situations, the schizophrenia patients feel as if they were stagnated while taking the medication, experiencing the maintenance of the clinical conditions of the disorder, without progresses and relapses.

*It's just that it always continues like that. [...] It doesn't get better but, if you don't take it *(the medication)*, it gets twice as bad. *(F3)

*He didn't have any relapse, but I don't see any progress either. But merely the fact that he didn't have any relapse is good I think! *(F9)

The option to abandon the pharmacological treatment can make the individuals more vulnerable to the exacerbation of previously controlled symptoms.

*If I didn't take it I got worse. When taking the medicine correctly I don't have this kind of thing... I hear voices, I see visions because I stop taking the medication.* (P6)

*Yes, I've already wanted to *(quit the medication)*sometimes. We insist blindly, you see? I had to get back to the same place, start over. *(P7)

The association between the non-adherence and the negative consequences is not an all-or-nothing phenomenon, as many patients adhere partially. Nevertheless, the literature has shown a significant impact even in mild degrees of non-adherence^(^ 6 ^,^ 23 ^)^. Evidence exists that non-adherence to antipsychotic medication use is related to relapses, more frequent hospitalizations, worse prognoses and higher costs^(^ 5 ^,^ 23 ^-^ 24 ^)^.

The consequences of the choices the patients have made in advance represent experiences they accumulate and consider when assessing the present situation and making new decisions, as human beings act in the present predominantly influenced by what is currently happening, but past experiences are applied in the action, according to the memories the individuals evoke^(^ 25 ^)^. Thus, like in a labyrinth, the schizophrenia patients keep on searching for a way out of the dilemma of "LIVING WITH A HELP THAT BOTHERS".

The experiences the patients accumulate and their future perspective provoked reflections about their behavior towards the medication treatment. Studies undertaken in distinct contexts certify that the improvement of symptoms, the prevention of relapses, the possibility of leading a normal life and the hope for the future favor the treatment adherence^(^ 13 ^,^ 15 ^)^.

## Conclusion

The act of taking a medication daily may seem routine and simple, but the experience is complex. For the schizophrenia patients, the effects of a drug are not limited to what the pharmacodynamics or pharmacokinetics can explain. As a result of the construction of meanings, medication use gains a larger dimension in the individuals' life.

The medication treatment affects the patients not only in terms of biochemical aspects, but also entails implications for their feelings and interactions, requires definitions, choices, attitudes, re-evaluations and redefinition of subsequent actions.

The schizophrenia patients perceive that, when following the medication treatment, they are "LIVING WITH A HELP THAT BOTHERS". Therefore, motivational interventions are recommended that address the patients' biased attitude towards the treatment, so as to favor the adherence.

The interaction with the client is an appropriate time to reconstruct meanings and, therefore, should be better explored as an instrument to promote treatment adherence and adaptive coping with the disorder.

The adherence behavior is complex and involves a wide range of external and subjective factors. To reach the decisive elements of treatment adherence, care strategies are recommended that are based on each client's reality and subjectivity.

One study limitation is the confirmation of the diagnosis by means of secondary sources (patient history and health team) and the restriction to schizophrenia patients under treatment at public health services within a delimited geographic territory. The theoretical model formulated in this study is valid for the study sample, within the context chosen. Nevertheless, it signals elements of the medication treatment compliance experience, which schizophrenia patients may share in different contexts.

It is highlighted that the theoretical model built in this study is not a set of closed, terminated and definitive premises. The emerging theory is continuously developing, with the possibility of deepening and expansion in other studies.
